# Interpretable pairwise distillations for generative protein sequence models

**DOI:** 10.1371/journal.pcbi.1010219

**Published:** 2022-06-23

**Authors:** Christoph Feinauer, Barthelemy Meynard-Piganeau, Carlo Lucibello

**Affiliations:** 1 Department of Computing Sciences, Bocconi University, Milan, Italy; 2 Bocconi Institute for Data Science and Analytics (BIDSA), Milan, Italy; 3 Laboratory of Computational and Quantitative Biology (LCQB) UMR 7238 CNRS, Sorbonne Université, Paris, France; 4 Department of Applied Science and Technologies (DISAT), Politecnico di Torino, Turin, Italy; University of Kansas, UNITED STATES

## Abstract

Many different types of generative models for protein sequences have been proposed in literature. Their uses include the prediction of mutational effects, protein design and the prediction of structural properties. Neural network (NN) architectures have shown great performances, commonly attributed to the capacity to extract non-trivial higher-order interactions from the data. In this work, we analyze two different NN models and assess how close they are to simple pairwise distributions, which have been used in the past for similar problems. We present an approach for extracting pairwise models from more complex ones using an energy-based modeling framework. We show that for the tested models the extracted pairwise models can replicate the energies of the original models and are also close in performance in tasks like mutational effect prediction. In addition, we show that even simpler, factorized models often come close in performance to the original models.

## Author summary

Complex neural networks trained on large biological datasets have recently shown powerful capabilites in tasks like the prediction of protein structure, assessing the effect of mutations on the fitness of proteins and even designing completely novel proteins with desired characteristics. The enthralling prospect of leveraging these advances in fields like medicine and synthetic biology has created a large amount of interest in academic research and industry. The connected question of what biological insights these methods actually gain during training has, however, received less attention. In this work, we systematically investigate in how far neural networks capture information that could not be captured by simpler models. To this end, we develop a method to train simpler models to imitate more complex models, and compare their performance to the original neural network models. Surprisingly, we find that the simpler models thus trained often perform on par with the neural networks, while having a considerably easier structure. This highlights the importance of finding ways to interpret the predictions of neural networks in these fields, which could inform the creation of better models, improve methods for their assessment and ultimately also increase our understanding of the underlying biology.

## 1 Introduction

Many different types of generative models for protein sequences have been explored, from pairwise models inspired by statistical physics [1–4] to more complex architectures based on neural networks like variational autoencoders [5–7], generative adversarial networks [8], autoregressive architectures [9, 10] and models based on self-attention [11]. While such models promise a rich field of applications in biology and medicine [12], the question of what information they extract from the sequence data has received less attention. This is, however, a very interesting field of research since especially the more complex models might extract non-trivial higher-order dependencies between residues. This in turn might reveal interesting biological insights.

Some recent works address this interpretability issue. In Ref. [13], the authors introduce the notion of *pairwise saliency* and use it to quantify the degree to which more complex models learn structural information and how this relates to the performance in the prediction of mutational effects. Ref. [14] instead constructs pairwise approximations to classifiers trained on categorical data and, among other results, show an example using protein sequence data.

We observe that the performance of many different models on tasks like the prediction of mutational effects is often similar even when using very different architectures and, in addition, is close to what simple, pairwise models achieve (see e.g. [9]). It appears natural to ask then how much of the predictive performance of the more complex models like variational autoencoders is due to higher-order interactions which are inaccessible to more simple models.

We therefore ask in this work how close trained neural network (NN) based models are to the manifold of pairwise distributions. To this end, we train two different architectures on protein sequence data. Interpreting these models as energy-based models [15], we present a simple way to extract pairwise models from them and analyze errors in energy between extracted and original models. We show that the subtle question of gauge invariance is important for this purpose and address this invariance ambiguity using different objective functions for the extraction. In addition, we show that even simpler models for which the probability distribution factorizes over positions in the protein can often come close to the performance of the original models after extraction.

Our work embeds itself into the field of *knowledge distillation* [16], where the goal is to extract simpler models from more complex models, improving the computational performance of the models and also increasing interpretability [17]. We also note, however, that higher-order interactions are not necessarily connected to a lack of interpretability: Restricted-Boltzmann Machines, for example, provide a way to model higher-order interactions in protein sequences that are still relatable to biological properties [18].

The main contributions of this paper are 1) we introduce a method for extracting independent and pairwise models for arbitrarily complex generative models for protein sequences, 2) we connect this task to the properties of gauge transformations and show that one can focus the extraction on different parts of the sequence space, 3) we show that pairwise models (and sometimes even independent models) are often good approximations for the original models in terms of the reconstruction of energies and also for tasks like mutational effect prediction.

The code and data for reproducing the experiments in this work are available at https://github.com/christophfeinauer/PairwiseDistillations.

## 2 Methods and data

While we present a more detailed description in the following sections, we describe in this section the general pipeline we use throughout the paper. Our goal is to analyse generative models for protein sequences trained on a multiple sequence alignment of homologous protein sequences. Denoting by *N* the length of the aligned sequences, these models define a probability distribution *p*(*s*), where *s* is an arbitrary sequence of amino acids of fixed length. One goal of generative modelling is to arrive at a *p*(*s*) which reflects faithfully the evolutionary constraints acting on the family, assigning high probability to sequences with high fitness and low probability to sequences with low fitness. A common benchmark for such models is the prediction of experimentally measured fitness values of mutated sequences. A successful model can then for example be applied when screening pathological mutations involved in human disease [2, 19].

The arguably simplest generative model is an *independent* model, where the probability factorizes over the positions in the protein, which is equivalent to saying that the log probability is a sum over independent terms including only a single position. Slightly more complex models are *pairwise* models, where the log probability is a function including terms depending on up to two positions. A central claim in recent literature is that models based on neural networks outperform simpler models because they can capture more complex constraints from the data, which would correspond to higher-order interactions (terms including more than two positions) in the log probability.

**Fig 1:**
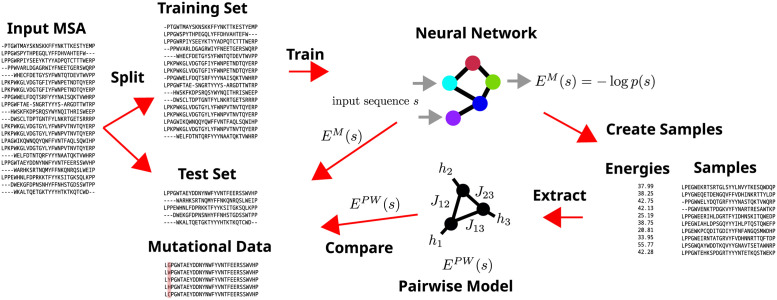
Pipeline. An overview of our train and test pipeline. Given an input MSA, we remove duplicated sequences and split into training and test set with a ratio of 9:1. We then train neural network models on the training set. After creating samples (either from the distribution induced by the neural network itself or from another distribution), we calculate the energy (negative log probability) of these samples from the neural network. We then use the samples and energies to extract a pairwise model (and also an independent model) and compare the original neural network model with the extracted in terms of their energies and their performance in mutational effect prediction.

In order to test and quantify this claim, we employ a simple pipeline (see Fig 1): We train neural network based generative models and test how well we can reproduce their distribution with independent and pairwise models. We do this by sampling sequences from either the uniform distribution or from the distribution induced by the original generative model itself, calculating the energies (negative log probabilities) of these sequences in the original generative models and then training a pairwise or independent model to reproduce these energies on the same sequences using a simple mean squared error loss. We then analyse how well these simpler models reproduce the probabilities of sequences on a test set and how well they perform in the task of the prediction of mutational effects when compared to the original models.

We provide a list of abbreviations for the various distributions and models we use in S1 Appendix.

### 2.1 Data and preprocessing

The training data for all models are aligned, homologous sequences of protein domains gathered in a multiple sequence alignment (MSA), where every row corresponds to a sequence of amino acids and every column to a position with homologous residues [20].

We denote a single amino acid sequence of length *N* as *s* = (*s*_1_, …, *s*_*N*_), where we identify every possible amino acid with a number between 1 and *q*, with *q* being the number of possible symbols (we use 20 amino acids and 1 alignment gap symbol, so *q* = 21). While we use this representation for the mathematical description and analysis, the implementations of the models use a one-hot encoding of the amino acids as inputs, where every amino acid is replaced by a vector of size *q* which contains zeros except at the position indicated by the integer corresponding to the amino acid. The input size for the models is thus a vector of size *Nq*.

The datasets we use are taken from [6]. Since some steps in our pipeline are computationally heavy, we selected 5 of the smaller of the 41 datasets used there. The datasets show a range of different properties with respect to the performances of different types of models (see Section 3.1). Every dataset consists of experimental fitness values for mutations in a target sequence and an MSA containing homologous sequences of the same target sequence. We use the datasets for the *BRCA1* tumor suppressor gene [21], the *GAL4* transcription factor [22], the small ubiquitin-like modifier *SUMO1* [23], the ubiquitination factor *UBE4B* [24] and the yes-associated protein *YAP1* [25]. When the dataset reported more than on experimental measurement we chose the same one as used in [9], see Table A2 in S1 Appendix for a list of experimental measurements used and the number of mutants in each dataset.

As the first step of preprocessing of the input MSAs, we replaced non-standard amino acids with a gap and removed all duplicated sequences from the datasets. We then partitioned the remaining sequences randomly into train and test sets, comprising 90% and 10% of the sequences respectively (see Table A1 in S1 Appendix for the number of sequences in the train and test sets).

**Fig 2:**
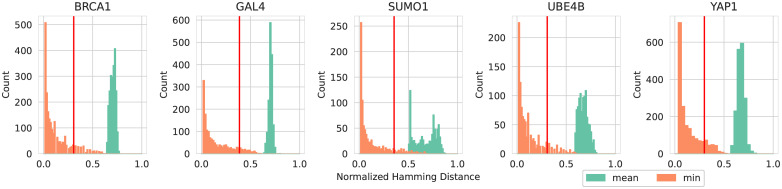
Normalized Hamming distance from test to train datasets. Shown are mean (green) and minimum (orange) distances from every test sequence to the sequences in the training set. The normalized Hamming Distance is the number of differing amino acids between two sequences, divided by the sequence length. The red bar indicates the distance at which the test set is divided in a ratio 9:1 with respect to the minimum distance to the training set.

While there are no identical sequences in the train and test sets due to the prior removal of duplicated sequences, for most families there is a fraction of the test sequences that have a Hamming distance of 1 to some sequence in the training set, see Fig 2. We note, however, that this should not pose a problem in the evaluation phase for the extracted models, since we do not extract models on the training sequences, but either from samples generated by the original models or from uniformly sampled sequences. In order to still control for possible effects on how well the extracted models reproduce the distribution of sequences in the test set with respect to the distance from the training set, we partition the test set further into two sub-datasets: One which contains the 10% of test sequences that are farthest from the training set in terms of the minimum Hamming distance and another one with the other 90% of sequences. In Fig 2 this cutoff is indicated by a red bar.

### 2.2 Original models

We train two different types of models on the datasets described in the last section: The autoregressive architecture presented in [9] (ArDCA), and a variational autoencoder (VAE) using the architecture from [5]. We refer to these models as the *original models* in order to distinguish them from the extracted models that are introduced later. For a given family, all models (including EVMutation, which we discuss in Sec.3.1) are trained on the same training MSA. All models apply a reweighting scheme, which aims at removing phylogenetic bias by giving a smaller weight in the objective function to sequences that have many similar sequences in the training set. We refer to the original publications for details on these.

ArDCA uses an autoregressive decomposition of the probability,

$$\begin{array}{c} p\left(s\right)=\prod_{i=1}^{N}p\left(s_{i}|s_{<i}\right), \end{array}$$ (1)

where

$$\begin{array}{c} p\left(s_{i}|s_{<i}\right)=\frac{\text{exp}(h_{i}\left(s_{i}\right)+\sum_{j=1}^{i-1}J_{ij}\left(s_{i},s_{j}\right))}{z_{i}\left(s_{<i}\right)}. \end{array}$$ (2)

Here, the *h* are parameters depending only on a single position and the *J* are parameters depending on pairs of positions. The *z*_*i*_(*s*_<*i*_) is a normalization depending on the part of the sequence preceding position *i*, which we denote by *s*_<*i*_. While the form is reminiscent of a pairwise model (see Sec. 2.3), the log probability log *p*(*s*) cannot be written as a sum of terms including only up to two positions, and therefore the model can in principle also include higher-order interactions. The model can be interpreted as a repeated application of the *softmax* operation common in classification using neural networks, predicting the next amino acid in the sequence given the previous ones.

Due to the autoregressive architecture, the probability *p*(*s*) and consequently the likelihood function can be calculated directly using Eq 2, where both the nominator and denominator are tractable. The training is done using the L-BFGS method [26], with an additional *L*2 regularization, see [9] for further details. We use the code provided by the authors for training.

The VAE we use follows the architecture in [5]. The decoder defines a probability distribution *q*_*ψ*_(*z*|*s*) over the latent representation *z* given the one-hot encoded input sequence *s* by using a multivariate Gaussian distribution with means *μ* = *W*^*μ*^*h*^*enc*^ + *b*^*μ*^, log variances log *σ*^2^ = *W*^*σ*^*h*^*enc*^ + *b*^*σ*^, and zero off-diagonal correlations. These are expressed in terms of the output of single layer: *h*^*enc*^ = tanh(*W*^*enc*^*s* + *b*^*enc*^). The weight matrices *W*^*enc*^, *W*^*μ*^ and *W*^*σ*^ together with the biases *b*^*enc*^, *b*^*μ*^, *b*^*σ*^ form *ψ*. The decoder *p*_Θ_(*s*|*z*) defines a factorized probability distribution over *s* given *z* given by *p*_Θ_(*s*|*z*) = *softmax*(*W*^*s*^*h*^*dec*^ + *b*^*s*^), with *h*^*dec*^ = tanh(*W*^*dec*^*z* + *b*^*dec*^). Here the weight matrices *W*^*dec*^, *W*^*s*^ and the bias vectors *b*^*dec*^, *b*^*s*^ form the set of parameters Θ. The prior over *z* is a standard Gaussian.

The VAE can be trained using the ELBO objective function, see [5] for details. We use the code of the authors of [5] to train the VAE models, which uses full-batch gradient descent on the ELBO objective and a weight decay regularization.

Since neither mutational effect prediction nor contact prediction is the central aim in [5], we test the VAE for a range of hyperparameters on all five datasets and assess the resulting performance on the task of mutational effect prediction, see Section 3.1 for details. The hyperparameters we test are the number of units in the hidden layers, the dimension of the latent representation and the weight decay setting. We then select a subset of these models for the application of the rest of the pipeline in Fig 1.

An assessment of the performance of the original models is done in Sec. 3.1.

### 2.3 Energy-based models

Using a framework based on Energy-based models (EBMs) [15], we can define a probability distribution *p*(*s*) over protein sequences by specifying the energy *E*_*θ*_(*s*), which is equal to the negative log probability up to a constant. The energy can be implemented for example by a neural network with weights and biases represented by *θ*. While the calculation of the exact probability

$$\begin{array}{c} p\left(s\right)=\frac{e^{-E_{\theta }\left(s\right)}}{Z_{\theta }}\;\text{with}\;Z_{\theta }=\sum_{s^{\prime }}e^{-E_{\theta }\left(s^{\prime }\right)} \end{array}$$ (3)

is intractable since the normalization constant *Z*_*θ*_ is a sum over *q*^*N*^ terms, numerous ways of training such models have been developed.

In this work, we use the fact that *any* probability distribution *p*(*s*) can be thought of as an EBM by defining *E*(*s*) ≔ −log *p*(*s*). We will use the term *energy* for both cases: when derived from a distribution *p*(*s*), and when given by an explicit energy function. While this formulation could be extended to models for sequences of varying length, we restrict ourselves in this work to sequences of fixed length.

### 2.4 Energy expansions and gauge freedom

We call *I* = {1, …, *N*} the set of all positions in the sequence *s* and *s*_*L*_ the subsequence consisting of amino acids at positions in *L* ⊆ *I*. Then, we can expand any energy *E*(*s*) in the form

$$\begin{array}{c} E\left(s\right)=\sum_{L\subseteq I}f_{L}\left(s_{L}\right), \end{array}$$ (4)

where *f*_*L*_ is a function depending only on the amino acids at positions at *L*, and the sum is over all subsets of *I*. We will use *f* for denoting the set of all *f*_*L*_ in the expansion. Models for which *f*_*L*_ = 0 for |*L*| > 2 are called *pairwise models* (or *Potts models*) and their energy can be written as a special case of Eq 4 as

$$\begin{array}{c} E^{pw}\left(s\right)=-\sum_{i=1}^{N}\sum_{j=i+1}^{N}J_{ij}\left(s_{i},s_{j}\right)-\sum_{i=1}^{N}h_{i}\left(s_{i}\right)-C, \end{array}$$ (5)

with *J* being commonly called couplings and *h* the fields [27]. Note that while the terminology is the same as for the parameters in ArDCA (see previous Sec.2.2), the parameters of ArDCA cannot be easily mapped to the parameters of a pairwise model. The constant *C* is typically not added to the model definition since it does not change the corresponding probabilities, but we keep it in order to be consistent with the generic expansion in Eq 4.

Models for which *f*_*L*_ = 0 for |*L*| > 1 are called *independent* or *profile* models [9]. Their energy function can be written as

$$\begin{array}{c} E^{ind}\left(s\right)=-\sum_{i=1}^{N}h_{i}\left(s_{i}\right)-C, \end{array}$$ (6)

which results in a factorized version of the probability distribution in Eq 3.

The expansion in Eq 4 is not unique, which means that given an energy *E*(*s*) it is possible to find different expansion parameters *f* for which Eq 4 holds. Therefore additional constraints must be imposed to fix the expansion coefficients (gauge fixing). It is for example trivial to rewrite the *pairwise* model in Eq 5 as a model with interactions only of order *N* by defining *f*_*I*_(*s*) = *E*(*s*) and *f*_*L*_ = 0 for |*L*| < *N*.

A common route is to impose the so-called *zero-sum* gauge [28], also called the *Ising* gauge, which aims to shift as much of the coefficient mass to lower orders as possible (see, e.g., Ref [14], where the authors use the term ‘Ising gauge’ and Section D.2 in S1 Appendix for details). This is intuitively sensible, since explaining as much of the variance as possible with low order coefficients seems to be a key element when trying to understand how complex the model is. However, we will show in the next section that the problem of gauge invariance is more subtle and important for understanding the structure of the fitness landscape induced by NN models.

### 2.5 MSE formulation for extraction

We formulate the problem of extracting pairwise and independent models from more general models by using a loss function L that measures the average mean squared error (MSE) in energies with respect to a distribution *D* over sequences. We call *E*^*M*^(*s*) the energy of the original model that we want to project onto the space of pairwise or independent models. We define the loss function over the parameters *J*, *h* and *C* on which the pairwise energy *E*^*pw*^(*s*) of Eq 5 implicitly depends as

$$\begin{array}{c} L\left(J,h,C\right)=E_{s∼D}[{\left(E^{M}\left(s\right)-E^{pw}\left(s\right)\right)}^{2}]. \end{array}$$ (7)

We minimize the loss function with respect to *J*, *h* and *C* and use the resulting pairwise model *E*^*pw*^ as an approximation to *E*^*M*^. For the independent model *E*^*ind*^(*s*) as defined in Eq 6, we use the same loss function but fix *J* to 0. Since independent models can be seen as special cases of pairwise models, we restrict the following discussion to pairwise models.

The distribution *D* is central in this formulation of the problem and is closely related to the question of gauge invariance. It can be shown that if *D* is the uniform distribution *U* over sequences, the minimizer of L(J,h,C) is equivalent to the pairwise part of *E*^*M*^ in the zero-sum gauge (see Section D in S1 Appendix for a proof). This means in reverse, that extracting the pairwise model using the zero-sum gauge is equivalent to minimizing the MSE in energy when giving all possible sequences equal weight. However, generative models trained on protein families are used only on a small region of the sequence space. By changing *D* it is possible to give more weight to these regions and construct a pairwise model that might be worse in replicating *E*^*M*^ globally, but better in regions of interest. This is equivalent to extracting the pairwise interactions in a different gauge of *E*^*M*^.

A natural candidate for *D* is the distribution induced by *E*^*M*^, leading to extracted models that aim to reproduce the original distribution well on typical sequences of that distribution. We denote this distribution by *M*. With this choice, the loss corresponds to an *f*-divergence (*f*(*t*) = *log*^2^(*t*)) in the unnormalized distribution space [29]. Notice also that for a trained model *E*^*M*^, one would expect this distribution to be close to the distribution of sequences in the training data.

One possible interpretation is that using different distributions *D*, one obtains extracted models corresponding to different ‘views’ of the original model: Using the uniform distribution *U*, we obtain an expansion that explains the original model distribution over the whole sequence space with minimal higher-order interactions. The extracted pairwise model then corresponds to the pairwise part in this expansion. Using the model distribution *M* itself, on the other hand, we obtain an expansion where the pairwise part explains as much of the energy variation on typical sequences from the model distribution itself, and the extracted pairwise model corresponds to this pairwise part. We show below that using the model distribution *M* has advantages when using the extracted pairwise model for reproducing the energies on the test sequences and also in many cases for mutational effect prediction.

Note that if the original model is in fact a pairwise model, then for any *D* with a sufficiently large support the minimizer of Eq 7 should correspond to the original model (up to a gauge transformation).

The method we use for minimizing the loss in Eq 7 depends on the distribution used for creating the samples. For the uniform distribution *U*, the exact minimizer of the loss can be calculated by taking averages over the energies of the original model *E*^*M*^(*s*) with *s* sampled from a uniform distribution, (see Section D.2 in S1 Appendix for a derivation). This leads to the sampling estimators

$$\begin{array}{cc} C & =-E_{s∼U}\left[E^{M}\left(s\right)\right]\\ h_{i}\left(a\right) & =-E_{s∼U}\left[E^{M}\left(s\right)|s_{i}=a\right]+C\\ J_{ij}\left(a,b\right) & =-E_{s∼U}\left[E^{M}\left(s\right)|s_{i}=a,s_{j}=b\right]+h_{i}\left(a\right)+h_{j}\left(b\right)+C, \end{array}$$

where $E_{s∼U}\left[E^{M}\left(s\right)\right]$ is the expectation of *E*^*M*^(*s*) when sampling *s* from the uniform distribution, $E_{s∼U}\left[E^{M}\left(s\right)|s_{i}=a\right]$ is the expectation of *E*^*M*^(*s*) when keeping *s*_*i*_ fixed to *a* and sampling the amino acids at the other positions from the uniform distribution and $E_{s∼U}\left[E^{M}\left(s\right)|s_{i}=a,s_{j}=b\right]$ is the expectation of *E*^*M*^(*s*) when fixing *s*_*i*_ to *a*, *s*_*j*_ to *b* and sampling the amino acids at the other positions from the uniform distribution. For independent models, only the first two equations are used.

For distributions *D* different from *U*, there is in general no simple sampling estimator for the parameters of the pairwise model *E*^*pw*^. Therefore, in the case where we set *D* = *M*, the distribution induced by *E*^*M*^(*s*), we resort to gradient descent on the loss in Eq 7. After preparing 10^7^ samples from the *M* distribution, we minimize the loss using the Adam optimizer [30] with a batch size of 10000. The samples in a batch are sampled individually from all available samples for every gradient descent step. Since not all amino acids might be observed in all positions in the samples from the model distribution *M*, we replace every sample in the batch individually with a sample from the *U* distribution with a probability of 1%. All parameters are initialized to 0. We keep an exponential moving average of the batch losses with a smoothing factor of 0.9 and stop optimizing if this average has not reached a new minimum within 1000 gradient descent steps. We chose 10^7^ samples based on the observation that minimizing the loss in Eq 7 can be formulated as solving a set of linear equations in the parameters of the extracted model (see Eq. 4 in S1 Appendix). While we cannot solve this set of equations directly for arbitrary distributions *D*, we aim to use a number of samples at least as large as the number of parameters we want to fit. The largest sequence length we have in our datasets is 77, which corresponds to about 1.3 × 10^6^ parameters. For independent models, we use the same method but discard the gradient of *J*.

### 2.6 Sampling from the original models

For both original models we create samples from the uniform distribution by sampling every amino acid (including the gap) at every position with an equal probability 1/*q*. For ArDCA, we obtain samples from the model distribution *M* by sampling amino acids sequentially using the expression in Eq 2. For the VAE models, we sample the latent factors *z* from a standard normal distribution and sample amino acids at every position given the probabilities as returned by the decoder.

For calculating the energies for sequences in ArDCA, we can again use the autoregressive decomposition in Eq 2. For the VAE models, we use importance sampling [31] with 5000 samples.

## 3 Results

### 3.1 Performance of original models

We train ArDCA and VAE models on the five different datasets described in Section 2.1. We assess their performance in terms of mutational effect prediction using the experimental values provided in the datasets, see Appendix Table A2 in S1 Appendix for a summary of experimental measurements used. These experimental values consist of quantitative fitness measures of sequences that contain mutations with respect to a wild-type sequence. Since these measures are different for the different datasets, we use the Spearman rank correlation between the energies of the mutants in the original models and the experimental values as an indicator of performance.

We also compare the performance of EVMutation [3], which is a method based on pairwise models, i.e., training an energy function as defined in Eq 5 directly on the training data. We retrain EVMutation using the code provided by the authors, where the training is based on the method of pseudolikelihoods (see [3] and [32] for details).

**Fig 3:**
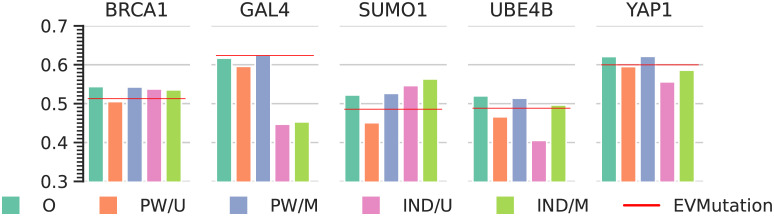
Spearman correlation with experimental data of original (O) and extracted models (PW/U, PW/M, IND/U, IND/M) for ArDCA. Shown is the Spearman rank correlation between the experimental data and the energies of the original model (O), the pairwise and independent models extracted using samples from a uniform distribution (PW/U and IND/U) and for the pairwise and independent models extracted using samples from the original model distribution (PW/M and IND/M).

For ArDCA, we use the code and hyperparameters optimized for generative modeling provided by the authors, using an *L*2 regularization of strength 0.01 for *J* and 0.0001 for *h* (values communicated by the authors of [9]). The performance in terms of the Spearman correlation is close to the ones reported in [9]: We report the Spearman correlation for the original ArDCA models in Fig 3 (the green bars). On four out of five datasets, ArDCA outperforms EVMutation and is roughly equal in performance on one dataset (GAL4).

For the VAE, we do not have hyperparmeters optimized for generative modeling. We therefore keep the general architecture of a single layer of hidden units in the decoder and encoder (same number of hidden units in encoder and decoder) and train for a series of different numbers of hidden units (40, 80, 100, 120, 140 and 160), different latent dimensions (5, 10, 20, 40, 80 and 120) and different settings for the weight decay strength (0.1, 0.05, 0.01, 0.005 and 0.001), using the code provided by the authors of [5]. We then assess the resulting 180 models for every dataset (so 900 models in total) in terms of their mutational effect prediction performance.

In Fig B1 in S1 Appendix we display the Spearman correlation values for all 900 models. From these results, it appears that the number of hidden units and the size of the latent dimension have minor effects on the performance. The weight decay, on the other hand has a more pronounced effect for the range we tested. Interestingly, this effect has opposite directions for different datasets: For example, a lower weight decay strength leads to generally better results for GAL4, while the best results for SUMO1 are obtained when using the strongest weight decay. This seems to correlate with the performance of independent models as reported in [9] (Fig 3 there). There the authors report that for example for SUMO1, an independent model trained directly on the training data performs *better* than EVMutation, which is based on a pairwise model. For GAL4 (called GAL1 in [9]), on the other hand, an independent model performs significantly worse than EVMutation. It is tempting to speculate that the stronger weight decay suppresses pairwise and higher-order interactions in the VAE, which in the case of SUMO1 seems to improve the performance and in the case of GAL4 decreases the performance. This would indicate that for SUMO1, some of the patterns the models capture in the data are not in agreement with the experimentally measured fitness values. We corroborate this finding in later sections.

**Fig 4:**
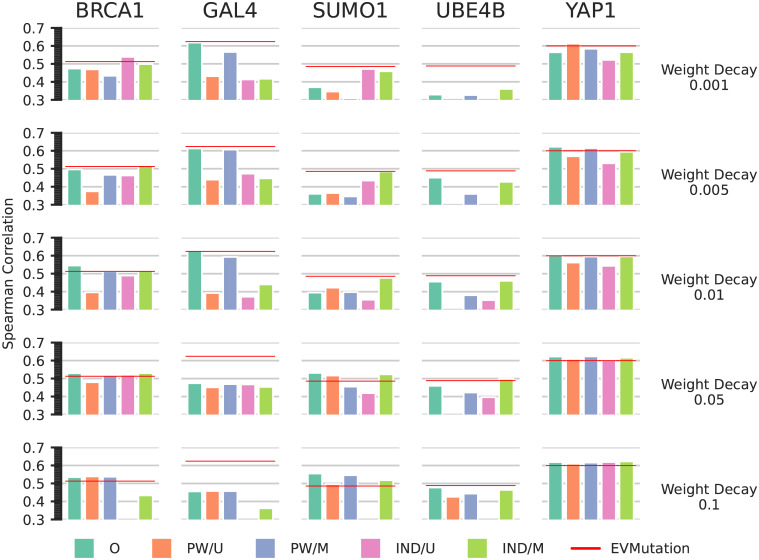
Spearman correlation with experimental data of original (O) and extracted models (PW/U, PW/M, IND/U, IND/M) for VAE models. Shown is the Spearman rank correlation between the experimental data and the energies of the original model (O), the energies of the pairwise and independent models extracted using samples from a uniform distribution (PW/U and IND/U), and the energies of the pairwise and independent models extracted using sequences sampled from the original model distribution (PW/M and IND/M). The rows correspond to different weight decay settings in the original model, as indicated on the right.

We note that for all datasets, we can find a VAE model that outperforms EVMutation in terms of mutational effect prediction. However, since we use the same experimental measurements to assess the performance of extracted models in later sections, choosing the best model based on these results might introduce biases. Since the number of hidden units and the size of the latent dimension seem to be less important, we fix these to 40 and 5 respectively, and run our pipeline for all five weight decay values. Within this subset of the trained models, there is always a model that performs as well or better than EVMutation (see green bars in Fig 4), except for UBE4B. On the same time, the performance is often very poor for specific weight decay settings: Setting for example strong weight decay of 0.1 for GAL4 reduces the Spearman correlation with the experimental data to well below 0.5, while EVMutation gives a correlation of more than 0.6.

### 3.2 Energy errors

**Fig 5:**
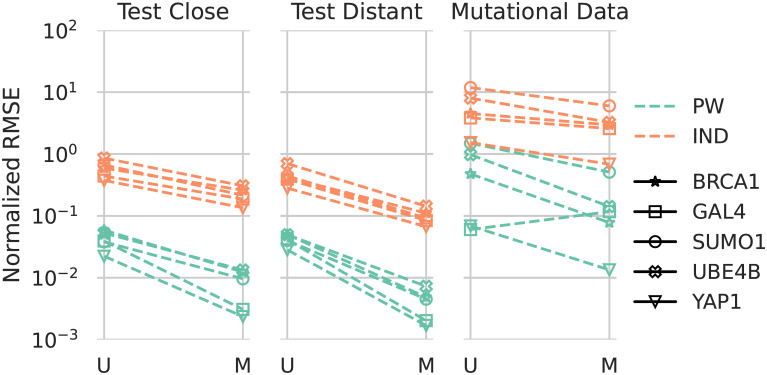
Errors in energies of the extracted pairwise and independent models with respect to the original ArDCA models. The three columns correspond to three different datasets: “Test Distant” corresponds to the 10% of sequences in the test set that have the largest distance to the training set in terms of minimum Hamming distance, “Test Close” to the remaining sequences. The right-most column corresponds to the sequences in the mutational datasets. The colors indicate whether the extracted model is an independent model (orange) or a pairwise model (green). Within every column, the left bar (U) corresponds to models extracted with samples from the uniform distribution, the right bar (M) to models extracted with samples from the distribution of the original models. The error shown is the normalized root-mean squared error (see Eq 8. Note the logarithmic scale.

In Fig 5 we show the error in the energies of extracted pairwise and independent models with respect to the energies in the original models for ArDCA. As described in Section 2, we use two different distributions *D* in Eq (7) for sampling the sequences used for the extraction of the pairwise models: The uniform distribution (U) and the distribution of the original generative model (M). We evaluate the error on the 10% of test sequences that are farthest from the training sequences in terms of the minimum Hamming distance (called ‘Test Distant’ in Fig 5), on the 90% remaining sequences (called ‘Test Close’ in Fig 5) and on the sequences from the mutational datasets.

The error in the plot is the root mean squared error, normalized by the range, i.e.,

$$\begin{array}{c} \text{Normalized}\;\text{RMSE}\;=\frac{\sqrt{\frac{1}{M}\sum_{m=1}^{M}{\left(E^{M}\left(s_{m}\right)-E^{pw}\left(s_{m}\right)\right)}^{2}}}{{\text{max}}_{m}\;E^{M}\left(s_{m}\right)-{\text{min}}_{m}\;E^{M}\left(s_{m}\right)}, \end{array}$$ (8)

where ${\left\{s_{m}\right\}}_{m=1}^{M}$ is the set of sequences on which we calculate the error, *E*^*M*^ is the energy of the original model, *E*^*pw*^ the energy of the extracted pairwise model and max_*m*_ *E*^*M*^(*s*_*m*_) and min_*m*_ *E*^*M*^(*s*_*m*_) are the maximum and minimum energies of the original model on the dataset.

The error for pairwise models is considerably smaller than for independent models, which is evidence that the original ArDCA models are indeed including at least pairwise interactions. In most cases, the error drops significantly when using the model distribution M for extraction instead of the uniform distribution U. This can be taken as evidence that the original models indeed include higher-order interactions and corroborates the idea that focusing on a specific part of the sequence space improves the quality of the extracted model on there. On the same time, the errors as percentages are relatively low for the extracted pairwise models: The errors are between 1% and 10% when using uniform samples and around 1% or below when using samples from M for extraction. This indicates that the ArDCA models are relatively close to pairwise models in the space around the training and test sequences.

**Fig 6:**
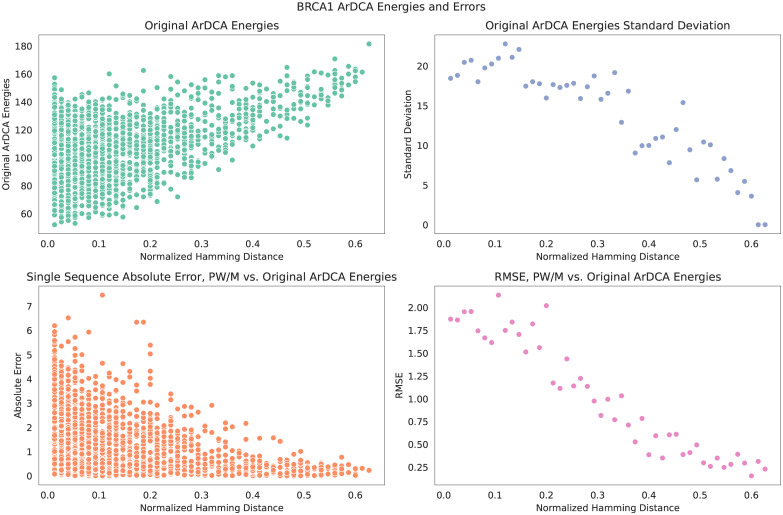
Energies and their standard deviations on BRCA1 test sequences. All plots share the abscissa, which shows the normalized Hamming distance of the test sequences to the closest sequence in the training set. *Upper left:* Energies of individual test sequences in the original ArDCA model. *Lower left:* Absolute errors on individual test sequences of a pairwise model extracted with sequences sampled from the original model distribution M with respect to the original energies. *Upper right:* Standard deviation of the energies in the original model at a given distance. *Lower right:* Root-mean-squared error on all test sequences at a given distance for a pairwise model extracted with sequences sampled from the original model distribution M with respect to the original energies.

Interestingly, the error on the test sequences distant from the training set is similar or smaller than the error on the test sequences closer to the training set. The largest error is on the sequences from the mutational dataset, which are very close to the training set. One possible explanation for this is found in Fig 6, where we plot for the test sequences of the BRCA1 dataset i) the energies of the test sequences in the original model; ii) their standard deviation at a given Hamming distance; iii) the absolute error on single sequence when comparing the energies from a model extracted with sequences sampled from the original model distribution; iv) the root-mean squared error, all in dependence of the normalized Hamming distance to the closest sequence in the training set. As can be seen in the upper right panel, there is an *inverse* relation between the standard deviation of energies in the original ArDCA model and the distance from the training set. The root-mean-squared error of the energies (the error defined in 8 without the denominator), shown in the lower right panel, closely follows this relation, meaning that the root-mean-squared error decreases on test sequences farther from the training set. We therefore speculate that the original model is more discriminative on sequences close to the training set, making it harder for the extracted pairwise model to reproduce the energy fluctuations there.

In Fig B3 in S1 Appendix we also show a scatter plot of the test sequences and the sequences of the mutational training set.

**Fig 7:**
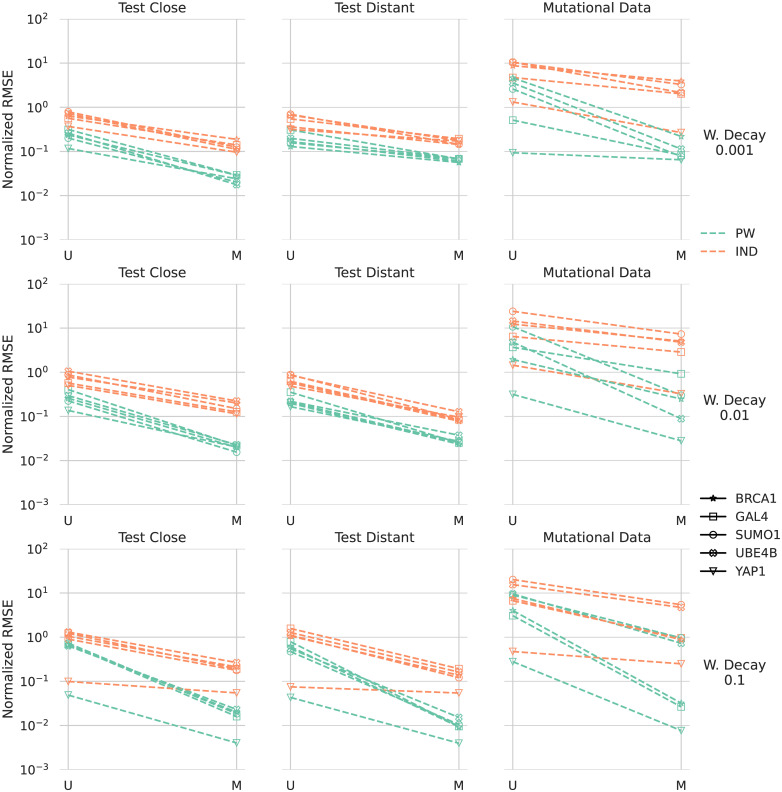
Errors in energies of the extracted pairwise and independent models with respect to the original VAE models. The three columns correspond to three different datasets: “Test Distant” corresponds to the 10% of sequences in the test set that have the largest distance to the training set in terms of minimum Hamming distance, “Test Close” to the remaining sequences. The right-most column corresponds to the sequences in the mutational datasets. The colors indicate whether the extracted model is an independent model (orange) or a pairwise model (green). Within every column, the left bar (U) corresponds to models extracted with samples from the uniform distribution, the right bar (M) to models extracted with samples from the distribution of the original models. The three rows correspond to different settings for weight decay during training. The error shown is the normalized root-mean squared error (see Eq 8. Note the logarithmic scale.

We show the errors for the VAE models with different weight decay settings in Fig 7. The general trend is very similar to the one described for ArDCA above, albeit with larger errors, indicating that the VAE models are less well described with pairwise models than ArDCA models. However, using sequences from the model distribution for extraction the error for pairwise models is mostly well below 10% on test sequences and, depending on the weight decay setting, often close to 1%. This indicates that when focusing on the part of the sequence space close to training and test sequences, the models can still be well approximated with pairwise models.

The performance of extracted pairwise and independent model seems to be closer together, which can be taken as evidence that the VAE models rely less on pairwise interactions. Also, the difference between extracted pairwise and independent models seems to increase when switching from using uniformly sampled sequences for extraction to sequences sampled from the original model distribution.

### 3.3 Comparing extracted couplings to EVMutation

**Fig 8:**
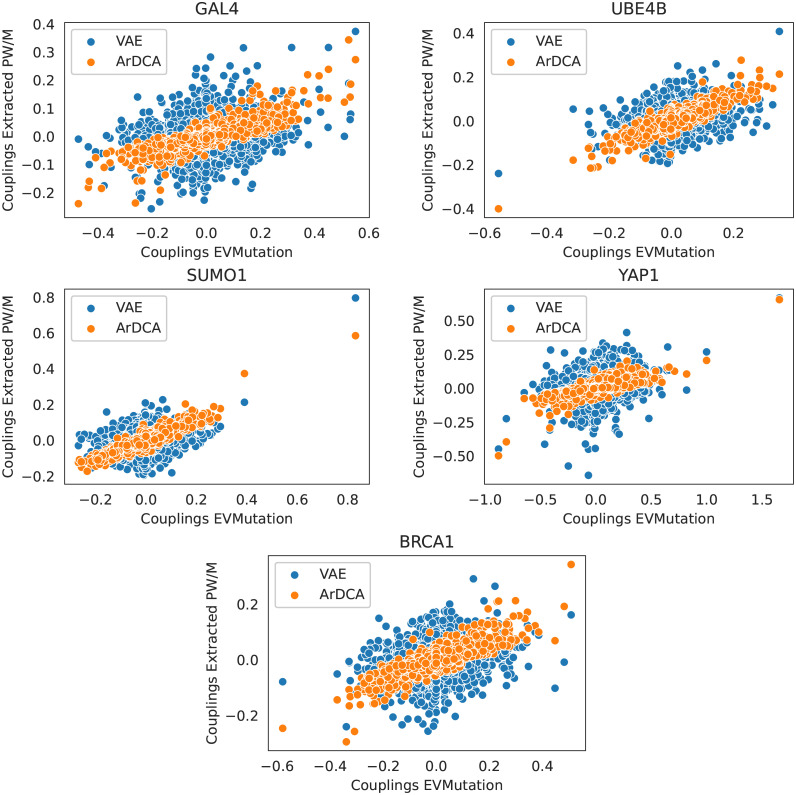
EVMutation couplings versus Couplings from PW/M Models extracted from ArDCA and VAE. Shown are scatterplots for all datasets of couplings as obtained from EVMutation after training versus couplings from pairwise models extracted using sequences sampled from the original model distribution, for ArDCA and VAE (weight decay setting 0.01). Given that the number of couplings is very large, we plot in all cases a subset of 5000 randomly chosen coupling pairs. Before plotting, all couplings were transformed to the zero-sum gauge.

Given that EVMutation is based on a pairwise model, we can directly compare the couplings from the extracted models to the ones obtained from EVMutation. In Fig 8 we plot couplings of pairwise models extracted with sequences sampled from the original model distribution against EVMutation couplings. For the VAE models, we chose original models with weight decay setting 0.01. As can be seen, the extracted ArDCA couplings follow the EVMutation couplings more closely than the extracted VAE couplings, although both are correlated. This can be seen as evidence that ArDCA models are closer to pairwise models directly trained on the input data. We note that this correspondence could likely be more pronounced for ArDCA by coordinating regularization strengths in EVMutation and ArDCA. This also suggests the possibility of using ArDCA with a subsequent extraction step as a training method for pairwise models, which can in general only be trained approximately for realistic sequence lengths. We leave this, however, for future research.

### 3.4 Mutational effect prediction using extracted models

The prediction of mutational effects is a typical field of application for the type of models analyzed in this work.

In Fig 3 we show the Spearman correlations between the experimental data and the energies in the original ArDCA models (O), the energies of pairwise and independent models extracted using samples from a uniform distribution (PW/U and IND/U) and the energies for models extracted using sequences sampled from the original model distribution (PW/M and IND/M). The red line indicates the performance of EVMutation, which is a pairwise model directly trained on the training data. The pairwise models extracted using sequences sampled from the original distribution reproduce the performance of ArDCA very closely, while pairwise models extracted using uniformly sampled sequences show a drop in performance. This corroborates the idea that while ArDCA models are not pairwise models in general, their characteristics on the part of the sequence space where they are typically used can be reproduced by a pairwise model.

Interestingly, for SUMO1 independent models extracted from the original models *outperform* the original models as well as extracted pairwise models. Since independent models are a special case of pairwise models (see Eq 5), this means that additional variability captured in the pairwise and original models hurts the performance in this case. For a possible explanation one can note that the available experimental fitness values are for sequences close to a wild-type sequence, and that the fitness landscape in this specific region might exhibit idiosyncrasies that are not mirrored in the fitness landscape at a larger scale or are even contradictory to it. Another possible explanation is experimental bias, which might systematically generate values for fitness proxies that are contradictory to the evolutionary patterns in the datasets used for training the models.

Another interesting case is BRCA1, where the original ArDCA models and the extracted independent and pairwise model perform similarly, and both outperform EVMutation. This suggests that one has to be careful when interpreting the relative performance of different model types: Considering only the values for the original ArDCA and EVMutation models, one might be tempted to conclude that ArDCA improves the predictions with respect to a pairwise model due to the capture of higher-order constraints. However, our results suggest that even an independent model can reach similar results. It is likely, therefore, that the differential performances in this case are less due to the intrinsic complexity of the model architecture but to specific choices of regularization and other training settings.

In order to test the robustness of these results, we run the pipeline a second time for the ArDCA models. Since the parameters of the original models and the fields and couplings of the extracted models are initialized to 0, the important factors in terms of stochasticity are the random seeds used for the splitting of the training and test sets, the random seeds used when sampling from the original models and the random seeds used for the stochastic gradient descent using the Adam optimizer when extracting the models. In Fig B2 in S1 Appendix we show the same plot as in Fig 3, but with all of these seeds set to a different value. The results show only minor variations.

In Fig 4 we show the results on mutation effect prediction for the VAE models for the different weight decay settings we tested. In most cases, the pairwise models extracted using sequences sampled from the original model distribution follow the performance of the original model more closely than pairwise models extracted using samples from the uniform distribution.

Interestingly, there is always a weight decay setting for the training of the original VAE model for which the independent model extracted using sequences sampled from the original model distribution performs as well or better than EVMutation, with the exception of GAL4. For SUMO1, the results corroborate the findings when using ArDCA: The original model performs better when a strong weight decay is used, and the extracted pairwise models follow this tendency. For low weight decay values, however, the independent model extracted using sequences sampled from the original model distribution performs significantly better than the original and the extracted pairwise models. As in the case of ArDCA, this can be taken as evidence that any interaction that the original model might capture in the data is incompatible with the experimental fitness values from the mutational dataset. We plot the fitness values against the model energies and the respective ranks in Figures B4–B8 in S1 Appendix.

### 3.5 Contact prediction

**Fig 9:**
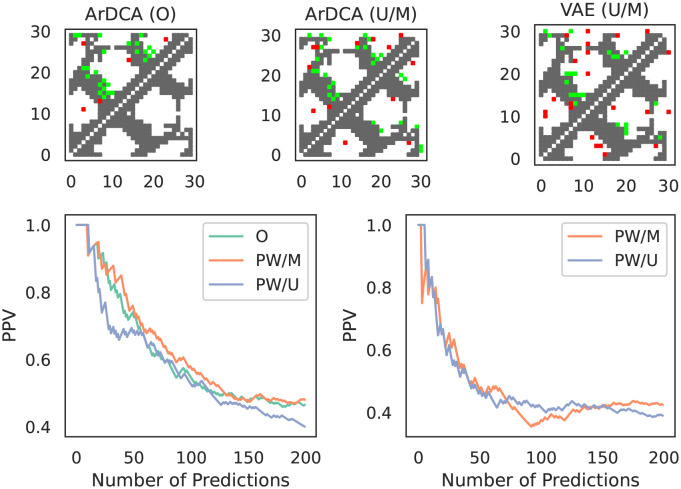
Contact prediction on YAP1 using extracted models. *Top Row*: Contact predictions vs. ground truth for the top *N* = 30 predicted contacts for models extracted from ArDCA and the VAE (weight decay setting 0.01). Horizontal and vertical axes show positions. True contacts are grey, true positives are green, and false positives are red. The upper parts show the contacts for models extracted with the uniform distribution, the lower parts show the same for models extracted with the original model distribution. The left-most plot shows the contact predictions for ArDCA from the original method in [9]. *Bottom Row:* PPV plots for the top 200 predictions for the original ArDCA model and the extracted pairwise model for ArDCA (left) and the VAE (right). The plots shows the fraction of true positives in dependence of the number of top predictions taken into account.

Given that we have the explicit couplings for the extracted pairwise models, we can use standard methods from this field to predict structural contacts [27, 32] (see Section C in S1 Appendix for the contact prediction pipeline and the PDBs used). We show the results in Fig 9.

For ArDCA, the contact predictions for the extracted pairwise models are largely the same, irrespective of which distribution the sequences used for extraction come from, and also very similar to the predictions from the original method. We note that the overall performance is not particularly good. This can be explained, however, by the fact that we did not use hyperparameters optimized for contact prediction for ArDCA, but hyperparameters optimized for generative modelling (see [9]).

The results for the pairwise model extracted from the original VAE models (trained with weight decay set to 0.01) are similar, although the overall performance for contact prediction is worse. This is in line with other recent results [13], where the authors show that VAE models can learn to predict mutational effects well but structural characteristics poorly.

## 4 Discussion

In this work, we provide evidence that the neural network based generative models for protein sequences analyzed by us can be approximated well by pairwise distributions in the part of the sequence space close to natural sequences, and in many cases even by the factorized distributions of independent models. The autoregressive architecture on which ArDCA is based seems to be closest to a pairwise model after training. For the VAE, the results seem to at least indicate that their pairwise projection is a very close approximation in the part of the sequence space in which they are typically used, close to the data manifold.

We cannot of course exclude that the neural network models tested by us do extract some meaningful higher-order interactions from the data, but the results seem to indicate that their effect is rather subtle. This suggests that the general strategy outlined in [33], where the pairwise part of the model is kept explicitly and an universal approximator is used for extracting higher-order interactions, might be promising. However, the current work also highlights that one has to be careful when ascribing improved performance of complex models to higher-order interactions. Apart from the fact that it is not trivial to define unambiguously what constitutes a higher-order interaction in the space of parameters due to gauge invariances, one also has to show that it is indeed the higher-order interactions that lead to the improved performance. Several works have highlighted that pairwise models, which are trained to reproduce the covariance in the data, are capable of reproducing data characteristics that are not used during training, for example three-point correlations [34]. At the same time, there are known cases where relatively clear higher-order interactions can be captured from the data and which can be included in the model, for example related to stretches of alignment gaps [35]. For the idea of combining a pairwise model with an universal approximator, this suggests that a promising approach is to regularize the combined model in way to give more prior importance to the pairwise part, and possibly to restrict the neural network to model few higher-order interactions.

Several interesting further lines of research suggest themselves. While the general idea of approximating a pairwise distribution over fixed-length sequences to models trained on unaligned data (like recent very large attention-based models [36]) seems to be ill-defined, the approach of extracting a pairwise model for a small part of the sequence space as highlighted in this work might still be feasible. Another interesting question is whether sparse higher-order interactions can be efficiently extracted from neural network based models. It is for example possible that methods like the Goldreich-Levin algorithm [37] might be adapted for pseudo-boolean functions based on generative models for protein sequence data.

## Supporting information

S1 Appendix Additional figures, mathematical derivations and a list of abbreviations. (PDF)
