# Supplementary material for: Interpretable pairwise distillations for generative protein sequence models
Source: PLoS Comput Biol. 2022 Jun 23;18(6):e1010219. doi: 10.1371/journal.pcbi.1010219 (PMC9258900; doi:10.1371/journal.pcbi.1010219)
Supplement: S1 Appendix — (PDF) [file pcbi.1010219.s001.pdf]

Appendix: Interpretable Pairwise Distillations for Generative Protein Sequence Models

Christoph Feinauer
Department of Decision Sciences
Bocconi Institute for Data Science and Analytics (BIDSA) Bocconi University, Milan, Italy
christoph.feinauer@unibocconi.it

Barthelemy Meynard-Piganeau
Laboratory of Computational and Quantitative Biology (LCQB) UMR 7238 CNRS - Sorbonne Université, Paris, France
Department of Applied Science and Technologies (DISAT), Politecnico di Torino, Torino, Italy
barthelemy.meynard@polytechnique.edu

Carlo Lucibello
Department of Decision Sciences
Bocconi Institute for Data Science and Analytics (BIDSA) Bocconi University, Milan, Italy
carlo.lucibello@unibocconi.it

June 15, 2022

A Data and Preprocessing

In the following we report the properties of the datasets used.

Dataset	Length	Sequences	Unique Sequences	Train Sequences	Test Sequences
BRCA1	75	39396	21639	19476	2163
GAL4	62	22985	15833	14250	1583
SUMO1	76	21695	8719	7848	871
UBE4B	75	16478	10248	9224	1024
YAP1	30	86353	17953	16158	1795

Table A1: Length and number of sequences used for all 5 datasets

Dataset	Measurement	Number of Mutants
BRCA1	function_score	494
GAL4	SEL_A_24h	1104
SUMO1	screenscore	1404
UBE4B	log2_ratio	603
YAP1	linear	313

Table A2: Characteristics of experimental datasets. The 'Measurement' column indicates which measurement was taken, where the names correspond to the ones used in the supplemental material of [1].

B Additional Results

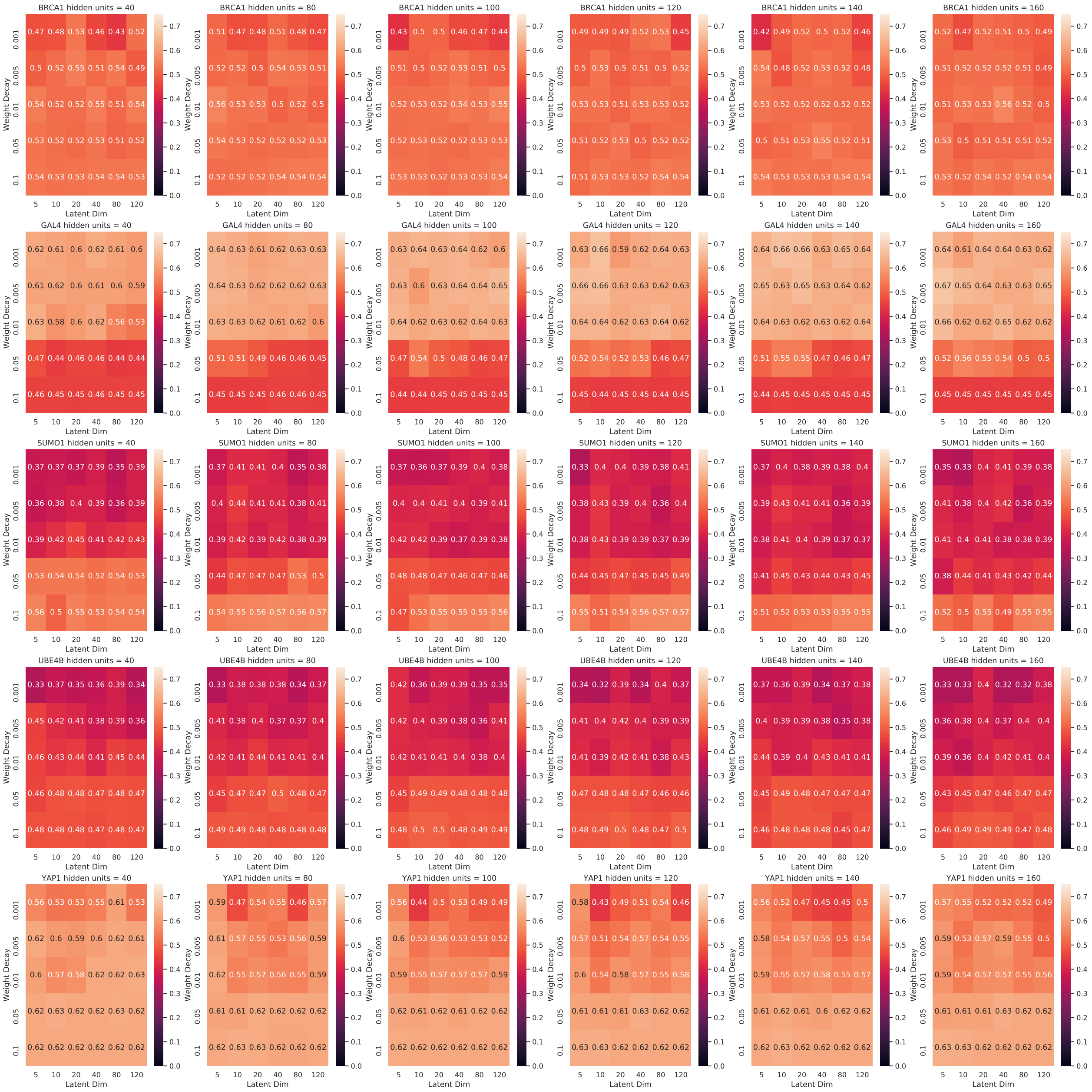

Figure B1: Spearman correlation with experimental values of VAE models trained with different hyperparameters. Every row corresponds to a dataset, every column to a different number of hidden units in the VAE encoders and decoders. Within every subplot, the rows correspond to different settings for the weight decay strength and the coulmns to different sizes for the latent dimension. The colors follow the Spearman correlation (the lighter the higher).

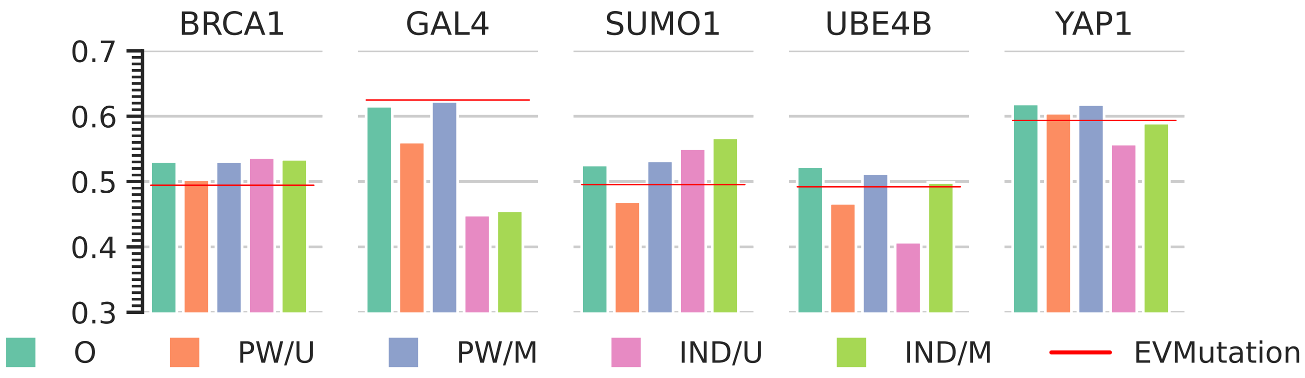

Figure B2: **Independent reproduction of main text Fig. 7: Spearman Correlation with experimental data of original (O) and extracted models (PW/U, PW/M, IND/U, IND/M) for ArDCA**. Shown is the Spearman rank correlation between the experimental data and the energies of the original model (O), the pairwise and independent models extracted using samples from a uniform distribution (PW/U and IND/U) and for the pairwise and independent models extracted using samples from the original model distribution (PW/M and IND/M). The random seeds changed for this reproduction are the seed used for the train-test split, the sampling of the sequences from the original models and the stochastic gradient descent using the Adam optimizer when extracting the model.

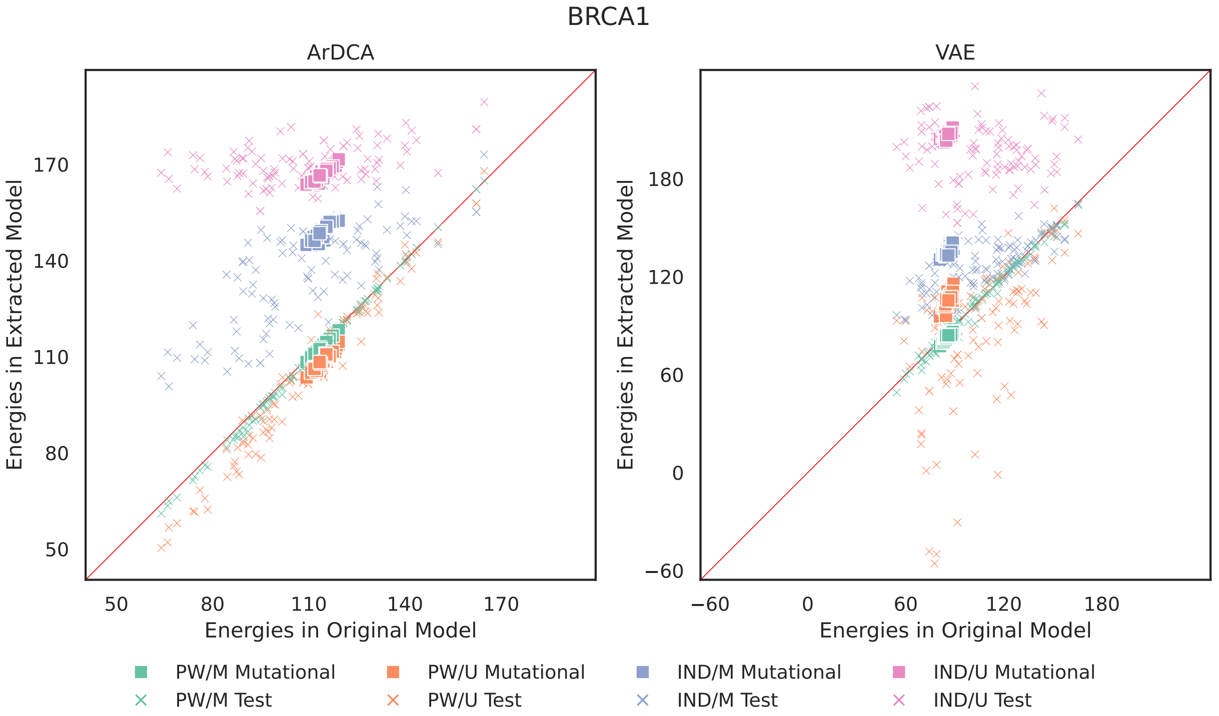

Figure B3: **Scatterplot Energies on BRCA1 Test Set** Shown are energies in the original models versus energies in the extracted models on the sequences in the test set (crosses) and the sequences from the mutational datasets (squares). The points for the test sequences correspond to 100 randomly chosen sequences. The VAE model used has 40 hidden units, a latent representation of size 5 and a weight decay setting of 0.01.

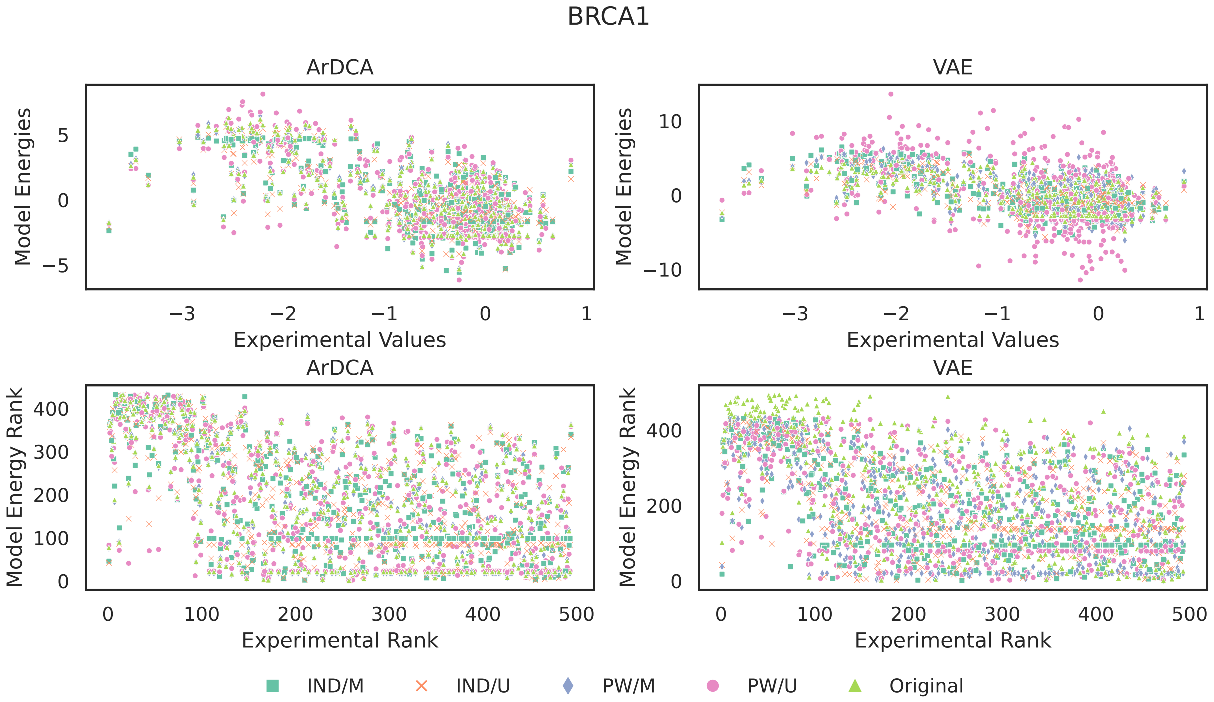

Figure B4: **Scatterplot Ranks and Energies on Sequences in Mutational Datasets for BRCA1** Shown are energies versus experimental fitness (upper two panels) and ranks of energies versus ranks of experimental fitness (lower two panels) for original and extracted models. Note that energies are negatively proportional to the log probability, so lower energy means higher probabilities. The VAE model used has 40 hidden units, a latent representation of size 5 and a weight decay setting of 0.01. If the number of mutants in the dataset was larger than 500, the plot shows data for a random subset of 500 mutants. The energies were normalized to have 0 mean for all models independently.

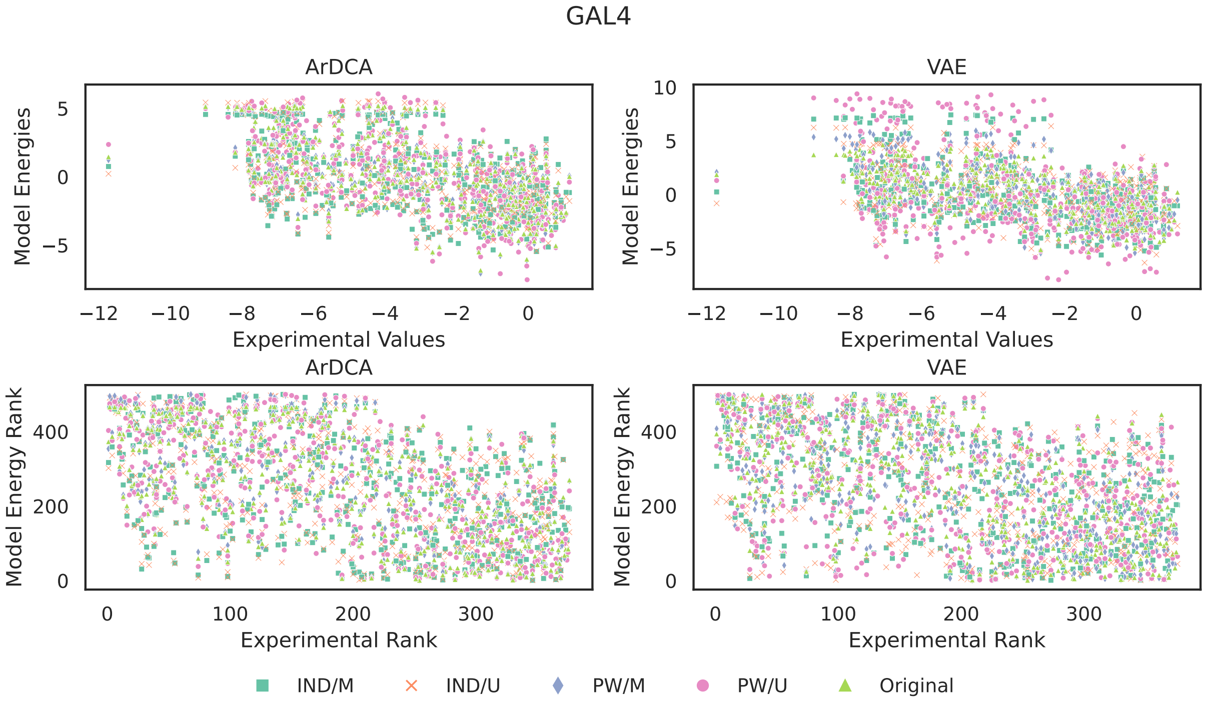

Figure B5: **Scatterplot Ranks and Energies on Sequences in Mutational Datasets for GAL4** Shown are energies versus experimental fitness (upper two panels) and ranks of energies versus ranks of experimental fitness (lower two panels) for original and extracted models. Note that energies are negatively proportional to the log probability, so lower energy means higher probabilities. The VAE model used has 40 hidden units, a latent representation of size 5 and a weight decay setting of 0.01. If the number of mutants in the dataset was larger than 500, the plot shows data for a random subset of 500 mutants. The energies were normalized to have 0 mean for all models independently.

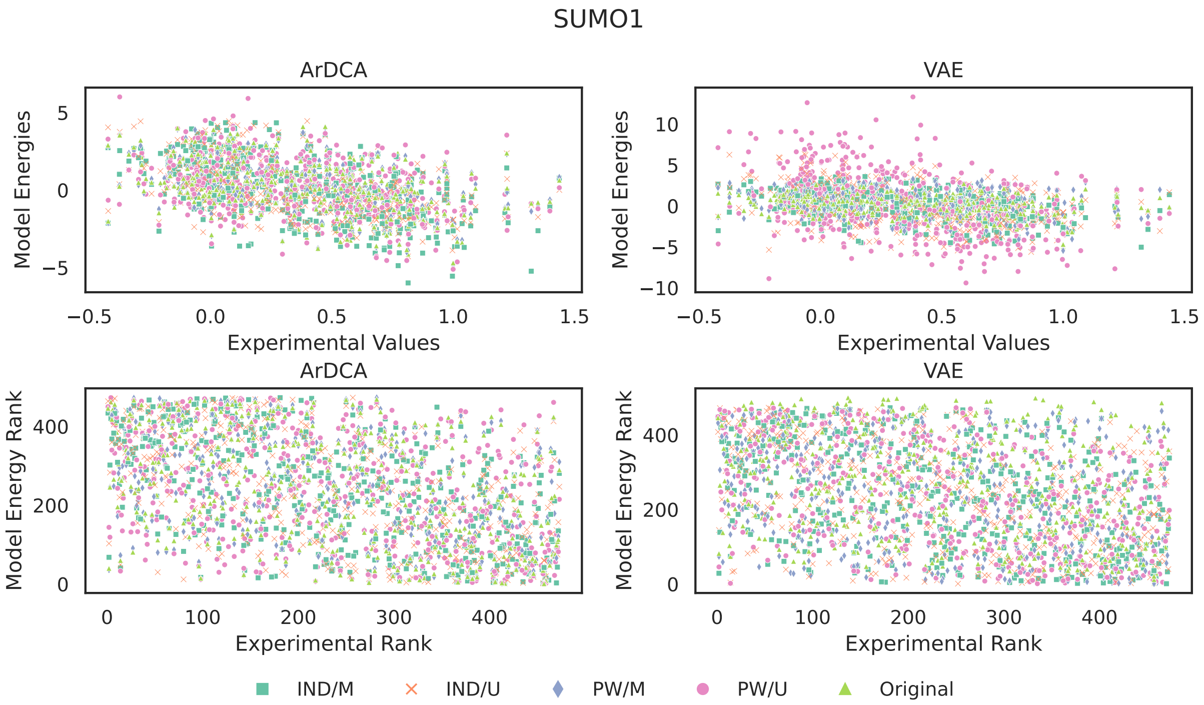

Figure B6: **Scatterplot Ranks and Energies on Sequences in Mutational Datasets for SUMO1** Shown are energies versus experimental fitness (upper two panels) and ranks of energies versus ranks of experimental fitness (lower two panels) for original and extracted models. Note that energies are negatively proportional to the log probability, so lower energy means higher probabilities. The VAE model used has 40 hidden units, a latent representation of size 5 and a weight decay setting of 0.01. If the number of mutants in the dataset was larger than 500, the plot shows data for a random subset of 500 mutants. The energies were normalized to have 0 mean for all models independently.

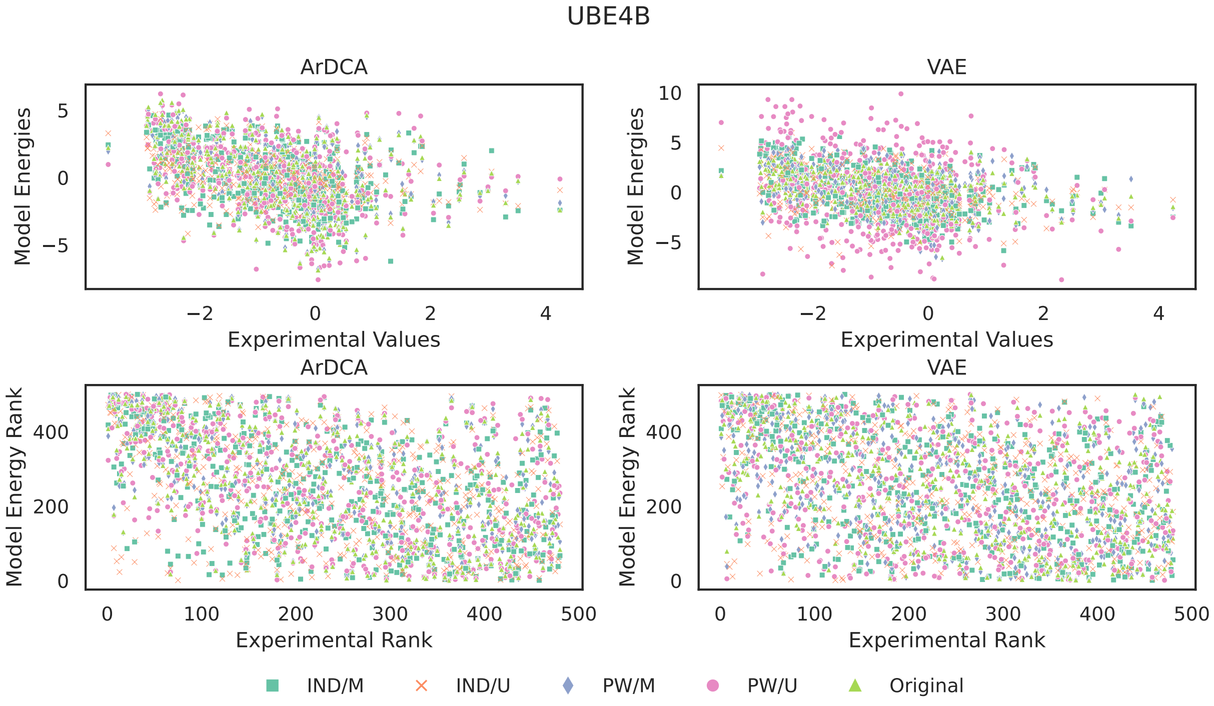

Figure B7: **Scatterplot Ranks and Energies on Sequences in Mutational Datasets for UBE4B** Shown are energies versus experimental fitness (upper two panels) and ranks of energies versus ranks of experimental fitness (lower two panels) for original and extracted models. Note that energies are negatively proportional to the log probability, so lower energy means higher probabilities. The VAE model used has 40 hidden units, a latent representation of size 5 and a weight decay setting of 0.01. If the number of mutants in the dataset was larger than 500, the plot shows data for a random subset of 500 mutants. The energies were normalized to have 0 mean for all models independently.

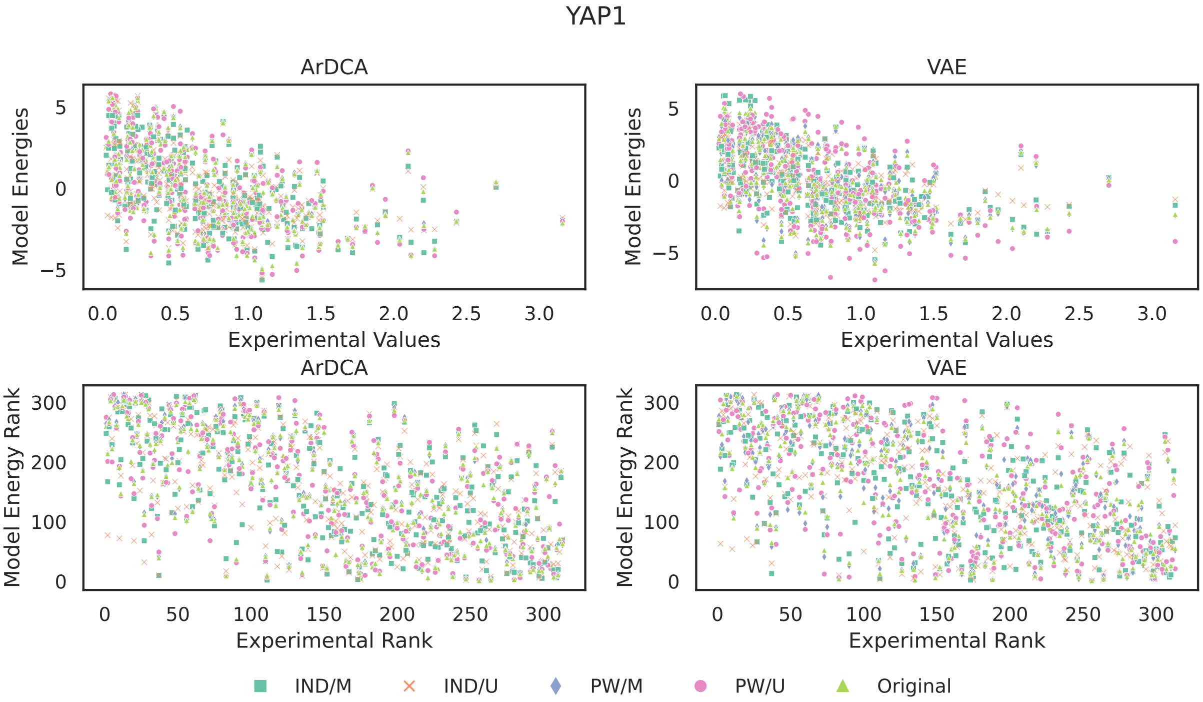

Figure B8: **Scatterplot Ranks and Energies on Sequences in Mutational Datasets for YAP1** Shown are energies versus experimental fitness (upper two panels) and ranks of energies versus ranks of experimental fitness (lower two panels) for original and extracted models. Note that energies are negatively proportional to the log probability, so lower energy means higher probabilities. The VAE model used has 40 hidden units, a latent representation of size 5 and a weight decay setting of 0.01. If the number of mutants in the dataset was larger than 500, the plot shows data for a random subset of 500 mutants. The energies were normalized to have 0 mean for all models independently.

C Contact Prediction

We use standard methods for contact prediction from pairwise models, following mainly [2]. We transform the extracted pairwise models into the zero-sum gauge and calculate the Frobenius norm of the $q-1 \times q-1$ submatrices J_{ij} corresponding to the pair of positions i and j (we do not sum over gap states, hence $q-1$ instead of q). We apply the *average-product correction* [3] and sort the positions pairs by the resulting score, excluding pairs for which $abs(i-j) < 5$. We map PDB 1PIN:A [4] to the MSA and use it to differentiate contacts from non-contacts (8 Å, Heavy-Atom criterion [5]).

D Zero-Sum Gauge

In the following we prove that the pairwise model E^{pw} corresponding to the minimizer of main text Eq. 7 is equivalent to the pairwise part of E^M in the zero-sum gauge when using the uniform distribution D for extraction.

D.1 Notation

We denote by $\mathcal{A} = \{1, .., q\}$ the (numeric) alphabet of the q possible amino acids. The terms $f_L : \mathcal{A}^{|L|} \to \mathcal{R}$ in the general expansion in main text Eq. 4 are functions mapping sequences of amino acids of length $|L|$ to a real number, where $L \subseteq I = \{1, \ldots, N\}$ is a subsequence of positions. In this notation, the pairwise model we train using the loss in main text Eq. 7 can be written as

$$E^{pw}(s) = \sum_{i=1}^{N} \sum_{j=i+1}^{N} f_{ij}^{pw}(s_i, s_j) + \sum_{i=1}^{N} f_i(s_i) + f_\emptyset. \tag{1}$$

In main text Eq. 5 we use a different notation for the pairwise model, but in this Appendix we decide to keep all notations compatible with the generic expansion in main text Eq. 4. The notations can be connected by identifying $f_i^{pw}(a) := -h_i(a)$, $f_{ij}^{pw}(a,b) := -J_{ij}(a,b)$ and $f_\emptyset := -C$ for arbitrary amino acids a and b.

Equivalently we define $f_L^M : \mathcal{A}^{|L|} \to \mathcal{R}$ as the interaction coefficients between the sites belonging to the set of positions $L \subseteq I$ in E^M in a certain gauge.

We will use $f_L(a_L)$ in order to denote a specific interaction coefficient for a fixed sequence of amino acids a_L of length $|L|$, for both pairwise models and models with higher-order interactions. We will use f^{pw} to denote the set of all parameters of the pairwise model and f^M for the set of all parameters of the original model.

D.2 Zero-Sum Gauge

The zero-sum gauge is a reparameterization of the interaction coefficients which leaves the energy invariant (see also Ref. [6] who discuss this gauge, calling it the *Ising* gauge). In this gauge, if $|L| > 0$, summing $f_L(a_L)$ over any of the amino acids in a_L while keeping the others fixed is 0. It can be applied both to the parameters of the extracted pairwise model f^{pw} and the parameters f^M of the original model. Since the sum over an amino acid is proportional to the expectation of $f_L(a_L)$ when the corresponding amino acid is sampled uniformly, this condition can be written as

$$\mathbb{E}_{s\sim U}[f_L(s_L)|s_J = a_J] = 0 \quad \forall\, J \subset L, \tag{2}$$

where $\mathbb{E}_{s\sim U}[f_L(s_L)|s_J = a_J]$ is the expectation of $f_L(s_L)$ if the subsequence s_J is fixed to a_J. Any model can be transformed into the zero-sum gauge using the identity $f_L(a_L) = (f_L(a_L) - \hat{f}_L(a_L)) + \hat{f}_L(a_L)$ with

$$\hat{f}_L(a_L) := \sum_{J\subseteq L} (-1)^{|J|} \frac{1}{q^{|J|}} \sum_{a_J} f^L(a_L). \tag{3}$$

It is easy to show that $\hat{f}(a_L)$ satisfies the condition in Eq. 2 and that $f_{a_L}(a_L) - \hat{f}_{a_L}(a_L)$ contains only interactions of order strictly less than $|L|$. Therefore, any model can be transformed into the zero-sum gauge by first applying the transformation to the interaction coefficients at the highest order $N = |I|$. This will lead to interaction coefficients at order N that satisfy the condition in Eq. 2 and new interaction coefficients of order lower than N. These can be

absorbed in the interaction coefficients in the lower orders of the expansion. Repeating this procedure at $N-1$, then at $N-2$ etc. leads to a final model where all interaction coefficients of all orders satisfy the condition in Eq. 2.

Since the expansion of E^M has exponentially many interaction coefficients in general, this procedure has no practical use in our setting. However, in the next section we show that the lower orders of E^M in the zero-sum gauge representation can be extracted with a simple sampling estimator.

D.3 Proof of Equivalence of Minimizer of Loss and Zero-Sum Gauge

The partial derivative of the loss in main text Eq. 7 with respect to a parameter $f_L^{pw}(a_L)$ in the pairwise model (note that $|L| \leq 2$ in this case) can be written as

$$\frac{\partial \mathcal{L}(f^{pw})}{\partial f_L^{pw}(a_L)} = 2\, \mathbb{E}_{s\sim U}\left[\left(E^{pw}(s) - E^M(s)\right) \frac{\partial E^{pw}(s)}{\partial f_L^{pw}(a_L)}\right]. \tag{4}$$

Setting the gradient to 0 leads to

$$\mathbb{E}_{s\sim U}[E^M(s)|s_L = a_L] = \mathbb{E}_{s\sim U}[E^{pw}(s)|s_L = a_L] \quad \forall L : |L| \leq 2 \tag{5}$$

which means that the minimisation of the loss with respect to the parameters of the pairwise model is equivalent to fitting the conditional expectation of the energy under uniform distribution up to the second order of the expansion.

Since the loss in main text Eq. 7 is invariant with respect to a gauge change in the pairwise model E^{pw}, we can assume without loss of generality that we extract the pairwise model in the zero-sum gauge representation. Using a hat to denote the parameters $\hat{f}^{pw}$ of the pairwise model in this specific gauge, it is easy to see from Eq. 1 and the condition in Eq. 2 that

$$\begin{aligned}
\mathbb{E}_{s\sim U}[E^{pw}(s)] &= \hat{f}_\emptyset^{pw} \\
\mathbb{E}_{s\sim U}[E^{pw}(s)|s_i = a] &= \hat{f}_i^{pw}(a) + \hat{f}_\emptyset^{pw} \\
\mathbb{E}_{s\sim U}[E^{pw}(s)|s_i = a, s_j = b] &= \hat{f}_{i,j}^{pw}(a,b) + \hat{f}_i^{pw}(a) + \hat{f}_j^{pw}(b) + \hat{f}_\emptyset.
\end{aligned}$$

Combining this with Eq. 5 we get at the minimum of the loss the conditions

$$\begin{aligned}
\mathbb{E}_{s\sim U}[E^{M}(s)] &= \hat{f}_\emptyset^{pw} \\
\mathbb{E}_{s\sim U}[E^{M}(s)|s_i = a] &= \hat{f}_i^{pw}(a) + \hat{f}_\emptyset^{pw} \\
\mathbb{E}_{s\sim U}[E^{M}(s)|s_i = a, s_j = b] &= \hat{f}_{i,j}^{pw}(a,b) + \hat{f}_i^{pw}(a) + \hat{f}_j^{pw}(b) + \hat{f}_\emptyset.
\end{aligned} \tag{6}$$

Similar to the pairwise model, we will use a hat to denote the parameters $\hat{f}^M$ of the model E^M in the zero-sum gauge. While the corresponding expansion

$$E^M(s) = \sum_{L\subseteq I} \hat{f}_L^M(s_L)$$

has interaction coefficients of all orders, we can again use the conditions in Eq. 2 to arrive at

$$\begin{aligned}
\mathbb{E}_{s\sim U}[E^{M}(s)] &= \hat{f}_\emptyset^{M} \\
\mathbb{E}_{s\sim U}[E^{M}(s)|s_i = a] &= \hat{f}_i^{M}(a) + \hat{f}_\emptyset^{M} \\
\mathbb{E}_{s\sim U}[E^{M}(s)|s_i = a, s_j = b] &= \hat{f}_{i,j}^{M}(a,b) + \hat{f}_i^{M}(a) + \hat{f}_j^{M}(b) + \hat{f}_\emptyset.
\end{aligned}$$

Taking these relations together leads to the minimizer condition

$$\hat{f}_L^{pw} = \hat{f}_L^{M} \quad \forall L : |L| \leq 2$$

which means that the E^{pw} minimizing the loss in main text Eq. 7 is the pairwise part of E^M in its zero-sum gauge representation. Note that the loss is still invariant with respect to a gauge change in the extracted pairwise model, so the extracted model can be in any gauge representation.

We also note that Eqs. 6 can be used to estimate the coefficients of the extracted pairwise model directly using uniform samples and the corresponding energies from the original models in order to approximate the expectations.

E List of Abbreviations in Main Text

Abbreviations in Figures and Text

PW: Pairwise Model
IND: Independent Model
O: Original neural network model
M: Distribution induced by original neural network model
U: Uniform Distribution
PW/M: Pairwise model extracted using samples from the M distribution and energies from O
PW/U: Pairwise model extracted using samples from the U distribution and energies from O
IND/M: Independent model extracted using samples from the M distribution and energies from O
IND/U: Independent model extracted using samples from the U distribution and energies from O
Test Distant: 10% of test sequences with largest Hamming distance to the training set
Test Close: Test sequences not in 'Test Distant'

References

[1] Riesselman AJ, Ingraham JB, Marks DS. Deep generative models of genetic variation capture the effects of mutations. Nature methods. 2018;15(10):816–822.

[2] Ekeberg M, Lövkvist C, Lan Y, Weigt M, Aurell E. Improved contact prediction in proteins: using pseudolikelihoods to infer Potts models. Physical Review E. 2013;87(1):012707.

[3] Dunn SD, Wahl LM, Gloor GB. Mutual information without the influence of phylogeny or entropy dramatically improves residue contact prediction. Bioinformatics. 2008;24(3):333–340.

[4] Ranganathan R, Lu KP, Hunter T, Noel JP. Structural and functional analysis of the mitotic rotamase Pin1 suggests substrate recognition is phosphorylation dependent. Cell. 1997;89(6):875–886.

[5] Morcos F, Pagnani A, Lunt B, Bertolino A, Marks DS, Sander C, et al. Direct-coupling analysis of residue coevolution captures native contacts across many protein families. Proceedings of the National Academy of Sciences. 2011;108(49):E1293–E1301.

[6] Zamuner S, Rios PDL. Interpretable Neural Networks based classifiers for categorical inputs. arXiv preprint arXiv:210203202. 2021;.
